# Identification and Functional Prediction of Long Non-Coding RNAs in Dilated Cardiomyopathy by Bioinformatics Analysis

**DOI:** 10.3389/fgene.2021.648111

**Published:** 2021-04-16

**Authors:** Yu-Xiao Chen, Jie Ding, Wei-Er Zhou, Xuan Zhang, Xiao-Tong Sun, Xi-Ying Wang, Chi Zhang, Ni Li, Guo-Feng Shao, Shen-Jiang Hu, Jian Yang

**Affiliations:** ^1^The First Affiliated Hospital, Zhejiang University School of Medicine, Hangzhou, China; ^2^Ningbo Medical Center Lihuili Hospital, Ningbo, China

**Keywords:** dilated cardiomyopathy, long non-coding RNAs, heart failure, transcript expression analyses, molecular targeted therapy

## Abstract

Dilated cardiomyopathy (DCM) is a relatively common cause of heart failure and the leading cause of heart transplantation. Aberrant changes in long non-coding RNAs (lncRNAs) are involved in DCM disorder; however, the detailed mechanisms underlying DCM initiation and progression require further investigation, and new molecular targets are needed. Here, we obtained lncRNA-expression profiles associated with DCM and non-failing hearts through microarray probe-sequence re-annotation. Weighted gene co-expression network analysis revealed a module highly associated with DCM status. Then eight hub lncRNAs in this module (FGD5-AS1, AC009113.1, WDFY3-AS2, NIFK-AS1, ZNF571-AS1, MIR100HG, AC079089.1, and EIF3J-AS1) were identified. All hub lncRNAs except ZNF571-AS1 were predicted as localizing to the cytoplasm. As a possible mechanism of DCM pathogenesis, we predicted that these hub lncRNAs might exert functions by acting as competing endogenous RNAs (ceRNAs). Furthermore, we found that the above results can be essentially reproduced in an independent external dataset. We observed the localization of hub lncRNAs by RNA-FISH in human aortic smooth muscle cells and confirmed the upregulation of the hub lncRNAs in DCM patients through quantitative RT-PCR. In conclusion, these findings identified eight candidate lncRNAs associated with DCM disease and revealed their potential involvement in DCM partly through ceRNA crosstalk. Our results facilitate the discovery of therapeutic targets and enhance the understanding of DCM pathogenesis.

## Introduction

Dilated cardiomyopathy (DCM) is a myocardial disorder defined by the presence of left ventricular or biventricular dilatation and left ventricular systolic impairment that are not explained by coronary artery disease or abnormal loading conditions (Elliott et al., 2008). In recent decades, due to early diagnosis and optimal implementation of pharmacological and non-pharmacological treatments, the long-term prognosis of DCM has improved, with up to 87% of patients now experiencing 8 years of survival free from death or heart transplantation (Merlo et al., 2014). However, DCM remains the leading cause of heart transplantation and accounts for 30 to 40% of all heart failure cases in large clinical trials (Haas et al., 2015). Therefore, further investigation of the mechanisms underlying DCM initiation and progression is required, and new molecular targets are urgently needed.

Long non-coding RNAs (lncRNAs) are defined as transcripts >200 nucleotides in length and lacking known protein-coding function (Batista and Chang, 2013), although still capable of participating in many fundamental biological processes and pathophysiological events. Emerging evidence reveals critical roles for lncRNAs in the development and progression of cardiovascular diseases (Lovren et al., 2012; Han et al., 2014; Micheletti et al., 2017; Zhang et al., 2018), and several studies report dysregulation of lncRNAs in association with DCM (Frade et al., 2016; Li et al., 2018a; Huang et al., 2019; van Heesch et al., 2019; Zhang et al., 2019; Zhou et al., 2019). However, the mechanisms associated with lncRNA-specific regulation of DCM initiation and progression remain vague.

In this study, we investigated lncRNA-expression profiles in heart tissues from DCM patients through microarray probe-sequence re-annotation. Using weighted gene co-expression network analysis (WGCNA), we constructed gene modules associated with DCM and identified hub lncRNAs, which were subjected to bioinformatics analysis to determine their subcellular localization and function in DCM disease. Our findings identified functional lncRNAs that could serve as potential therapeutic targets for DCM and provided useful insights into the mechanism and function of these lncRNAs.

## Materials and Methods

**FIGURE 1:**
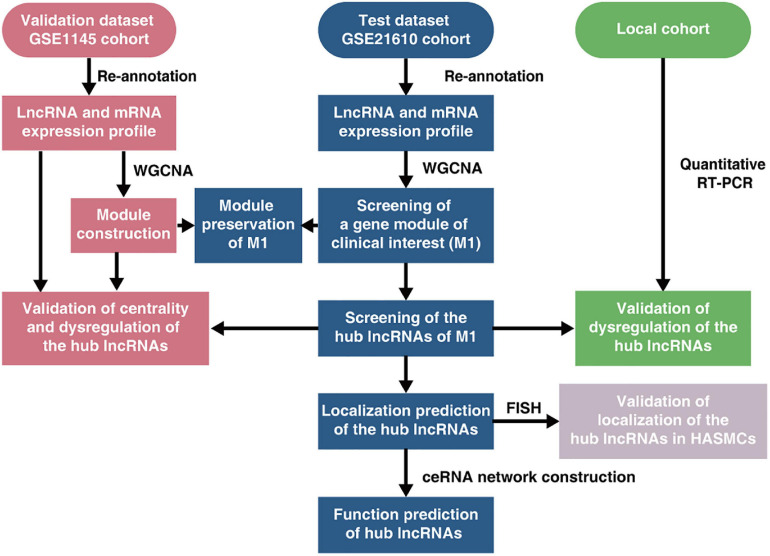
Schematic flow chart depicting the general steps taken in this research. ceRNA, competing endogenous RNA; FISH, fluorescence *in situ* hybridization; HASMCs, human aortic smooth muscle cells; lncRNA, long non-coding RNA; RT-PCR, reverse transcription-polymerase chain reaction; WGCNA, weighted gene co-expression network construction analysis.

A schematic flow chart depicting the general steps taken in this research is presented in Figure 1. And an expanded methods section is available in the Supplementary File.

### Data Preprocessing and lncRNA Re-Annotation

We systematically searched Gene Expression Omnibus (GEO) database and utilized GSE21610 as test dataset, GSE1145 as validation dataset. The selection criterion of dataset for test and validation was shown in the Supplementary File. Then the CEL format files of test dataset (GSE21610) and validation dataset (GSE1145) were downloaded from the GEO database. The GSE21610 cohort comprised 21 DCM patients and 8 non-failing donors, whereas the GSE1145 cohort comprised 12 DCM patients and 11 non-failing donors. And all the heart tissue used for microarray analysis was collected from left ventricle. Comprehensive clinical information of subjects enrolled in GSE21610 and GSE1145 were abstracted from the series matrix files provided in GEO database and corresponding published article, and presented in the Supplementary Table 1.

Then the raw probe-level intensity data in CEL format files were preprocessed using the robust multi-array average algorithm (Irizarry, 2003) for background adjustment, normalization, and summarization. Afterward, the probe-level intensity value was changed into probeset-level intensity value.

The probe-annotation sequences of HG-U133 Plus_2.0 were obtained from the official Affymetrix website. NCBI BLAST + 2.8.0 was used to align all probe-annotation sequences to the human long non-coding transcript sequences and human protein-coding transcript sequences from the GENCODE database. Results from sequence alignments were filtered using the following criteria: (1) the probe should perfectly hit a transcript (*E*-value < 2 × 10–6; query coverage: 100%; identity: 100%); (2) any probe perfectly hitting multiple targets was eliminated; and (3) transcripts matched by fewer than three probes were discarded. Probes were re-annotated according to the retained alignment result. Then each probeset was mapped to the corresponding lncRNA or mRNA. The probeset intensity was interpreted as lncRNA/mRNA expression, and used for further analysis.

### Differential Expression Analysis

Differentially expressed transcripts were identified using the “limma” R package (Ritchie et al., 2015) between disease and control samples. Only transcripts with an adjusted *P* < 0.05 were considered as differentially expressed transcripts.

### Gene Ontology Enrichment Analysis

Gene ontology (GO) is a standard vocabulary of functional terms and allows for coherent annotation of gene products. GO is structured in three parts: biological process, cellular component, and molecular function. Protein Analysis Through Evolutionary Relationships (PANTHER) (Mi et al., 2017) was used for GO analysis. The most characteristic GO and pathway terms ranking in the top five according to the “PANTHER overrepresentation test” function with statistical significance (*P* < 0.05) were extracted.

### Weighted Gene CoEexpression Network Construction

As suggested by the author of WGCNA, the top 25% varying genes were selected based on variance and then used to construct a co-expression network using the WGCNA R package (Langfelder and Horvath, 2008). We used the “step-by-step network construction and module detection” method and selected a soft threshold power (β = 14) to produce networks with a scale-free topology model fit that were >0.9. The topological overlap matrix was then transformed from the adjacency matrix, which organized hierarchical clustered genes into modules by average linkage. Finally, we merged similar modules with module eigengene (ME) distances were <0.2.

Modules that were significantly associated with DCM status and the hub lncRNAs in modules were determined as described previously (Langfelder and Horvath, 2008). Hub lncRNAs were identified based on a module membership (MM) value >0.75.

### Module Preservation Analysis

Module preservation between the test and validation datasets was assessed using the “modulePreservation” function of the WGCNA package (Langfelder et al., 2011). A composite preservation statistic Zsummary >10 suggested that there was strong evidence that the module of GSE21610 is preserved in GSE1145 (Langfelder et al., 2011).

### Assessing the Reproductivity of Hub lncRNAs in M1

To investigate the robustness of the hub lncRNAs screening results, a bootstrap analysis was used. First, to assess whether the results are robust to changes in input genes, we randomly re-sampled 90% of the initial input gene set 100 times. In each of the 100 bootstrap iterations, the co-expression network was recreated, and the hub lncRNAs were identified according to the following criteria: (1) these lncRNAs were assigned to the most significant DCM-associated module; (2) the MM values of these lncRNAs were above 0.75. Then we draw plots to visualize the bootstrap results. The red shading was used to illustrate whether the corresponding gene was hub lncRNA in corresponding iteration. And the consistency rates are calculated as the ratio of the number of the shaded cells to the total number of cells.

Next, to assess whether the results are robust to various WGCNA parameter values, we performed a parameter sweep over soft power, minModuleSize, deepSplit and CutHeight. We varied each parameter from 50 to 150%, while holding the other parameter values at their baseline values (soft power = 14, minModuleSize = 30, deepSplit = 2, and CutHeight = 0.2). Then co-expression networks were recreated, and the following analyses were the same as described in the paragraph above.

### Subcellular Localization Prediction

DeepLncRNA (Gudenas and Wang, 2018), lncLocator (Cao et al., 2018), and iLoc-lncRNA (Su et al., 2018) were used to predict the subcellular localization of lncRNAs. For genes having multiple transcripts, those displaying hits according to the Affymetrix microarray probesets were used for subcellular-localization prediction.

### Construction and Analysis of a Dysregulated lncRNA-Associated Competing Endogenous RNA Network

All lncRNAs and mRNAs in Module 1 (M1) were used to construct a competing endogenous RNA (ceRNA) network using the GDCRNATools (Li et al., 2018b) R package. The miRcode database (Jeggari et al., 2012) was used to collect predicted and experimentally validated microRNA (miRNA)-mRNA-interaction data, as well as miRNA–lncRNA-interaction data. Competing lncRNA–mRNA pairs were identified using the following criteria: (1) the lncRNA and mRNA must share a significant number of miRNAs (hypergeometric test *P* < 0.01); and (2) expression of lncRNA and mRNA must be positively correlated (Pearson’s correlation >0.7; correlation *P* < 0.01). These identified lncRNA-mRNA pairs were used to construct the ceRNA network, which was visualized using Cytoscape 3.6.1 software.

### Validation on GSE1145

After data preprocessing and lncRNA re-annotation on the GSE1145, the normalized intensity value of each gene was obtained. Then we applied the Student’s *t*-test directly on the intensity value of the eight hub lncRNAs to investigate whether these lncRNAs were dysregulated in DCM patients. The intensity value was also used as input data for receiver operating characteristic (ROC) analysis. Next, WGCNA was performed in the same way as described for GSE21610. Soft threshold power was set at 14, in which R2 was 0.81. Hub nodes of the Module (1) [M(1)] were identified based on a MM value > 0.75. Then all lncRNAs and mRNAs in M(1) were used to construct a ceRNA network. LncRNA-mRNA pairs were identified based on the same criteria mentioned above.

### Cell Culture and RNA Fluorescence *in situ* Hybridization

Human aortic smooth muscle cells (HASMCs) were purchased from the Academy of Sciences of China (China), and were cultured in Dulbecco’s Modified Eagle Medium (Hyclone, United States) containing 10% of fetal bovine serum. Cy3-labeled lncRNA probes were synthesized at Bersinbio Company (China). HASMCs were fixed for 10 min with 4% paraformaldehyde, and permeabilized in 0.1% Triton X-100 for 5 min. Then they were blocked in prehybridization buffer. Hybridization was carried out overnight at 37°C with fluorescence *in situ* hybridization (FISH) probe. Next day, cells were washed and stained with DAPI. All images were obtained with the Nikon A1R Confocal Microscope.

### Human Experiments

All human experiments were conducted in accordance with the Declaration of Helsinki. The protocol was approved by the Ethics Committee of the First Affiliated Hospital, Zhejiang University School of Medicine (No. 20191328). The participants provided their written informed consent to participate in this study.

The local cohort included nine non-failing control subjects and 12 DCM patients from the First Affiliated Hospital, Zhejiang University School of Medicine. The nine non-failing control subjects were mitral stenosis (MS) patients with echocardiographic normal left ventricular dimensions and normal heart function, and left ventricular papillary muscle tissue was collected from the MS patients at the time of mitral valve replacement. For 12 DCM samples, left ventricular myocardial tissue was collected from the DCM patients who underwent heart transplantation. All 21 samples were snap-frozen in liquid nitrogen after dissection and stored at −80°C until RNA extraction was performed.

### RNA Isolation and Quantitative Reverse Transcription-Polymerase Chain Reaction (RT-PCR)

Approximately 15–20 mg of frozen heart tissue, dissected free of fat, connective tissue, and large arteries and veins, was fully dissociated in RNAiso Plus (Takara, Japan). Then total RNA (1 μg) was extracted.

Reverse transcription was performed by adding RNA template, RNase-free water, and a master mix reagent (Takara, Japan), which includes PrimeScript RTase, RNase inhibitor, random 6mers, oligo dT primer, dNTPs, and reaction buffer. The reaction was carried out in a thermal cycler (Bio-Rad Laboratories, United States) for 15 min at 37°C followed by a heat inactivation of the reverse transcription enzyme for 5 s at 85°C.

Then quantitative PCR was performed in triplicate for the eight lncRNAs (FGD5-AS1, AC009113.1, WDFY3-AS2, NIFK-AS1, ZNF571-AS1, MIR100HG, AC079089.1, and EIF3J-AS1). The primers for detection of lncRNAs were produced by Sangon Biotech (China). All primers sequences are listed in Supplementary Table 2. 1 μl cDNA was combined with 5 μl TB Green Premix Ex Taq, 0.2 μl of 10 μM forward primer, 0.2 μl of 10 μM reverse primer and 3.6 μl of RNase-free water to a 10 μl reaction volume. The reaction was carried out on the Roche LightCycler 480 II machine (Roche, Switzerland) at 95°C for 30 s followed 40 cycles of 95°C for 5 s and 60°C for 30 s. Expression was quantified using 2^–ΔΔCt^ method and normalized to glyceraldehyde 3-phosphate dehydrogenase (GAPDH). The results were presented as fold change compared with control subjects.

### Statistical Analysis

Results were expressed as the mean ± standard deviation. Comparison between DCM and control donors was analyzed using an unpaired, two-sided Student’s *t*-test. Differences between DCM patients before and after left ventricular assist device (LVAD) support were analyzed by repeated measurement analysis of variance. ROC curves were established for discriminating DCM patients and control donors. Sensitivity and specificity were calculated according to standard formulas. The statistical software used was R (v.3.5.0).

## Results

### Differentially Expressed lncRNAs and mRNAs Profiles in DCM

After systematic searching GEO database using the keyword “dilated cardiomyopathy,” 2 eligible datasets (GSE21610 and GSE1145) were identified. GSE21610 was used as test dataset and GSE1145 was used as validation dataset.

**FIGURE 2:**
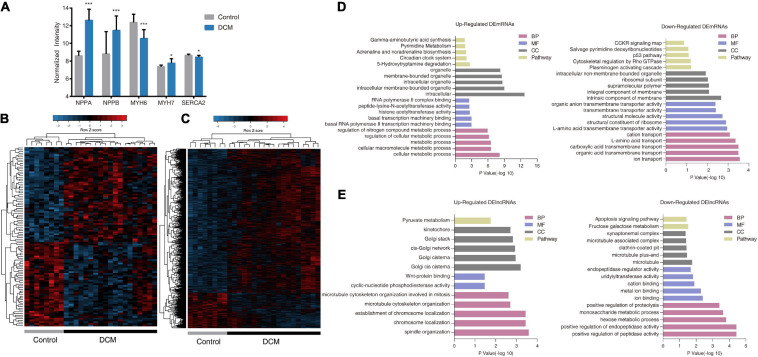
Transcriptome profiles of differentially expressed lncRNAs (DElncRNAs) and differentially expressed mRNAs (DEmRNAs) in dilated cardiomyopathy (DCM) patients and control donors in GSE21610. **(A)** Expression of various genes involved in heart failure in DCM patients and control donors. All values are mean ± SD. ***P < 0.001; *P < 0.05. **(B,C)** Heatmaps and unsupervised hierarchical clustering of DElncRNAs **(B)** and DEmRNAs profiles **(C)**. Panels show row Z scores. **(D,E)** The top five significantly enriched PANTHER Gene Ontology (GO) and pathway terms of up-regulated (left panels) and down-regulated (right panels) DEmRNAs **(D)** and DElncRNAs **(E)**. BP, biological process; MF, molecular function; CC, cellular component.

To obtain an initial understanding of transcriptional variations between DCM and non-failing hearts, test dataset GSE21610 (21 DCM patients and eight non-failing donors) was downloaded from the GEO database for advanced probe re-annotation of 3,629 lncRNAs and 16,070 mRNAs. Among these, we confirmed increased expression of NPPA, NPPB, and MYH7, as well as decreased expression of MYH6 and SERCA2 during quality assessment (Figure 2A).

We then identified 104 differentially expressed lncRNAs (DElncRNAs) and 1,867 differentially expressed mRNAs (DEmRNAs) between DCM patients and control subjects in GSE21610. We identified 55 lncRNAs and 1,498 mRNAs as upregulated, whereas 49 lncRNAs and 369 mRNAs were downregulated. Unsupervised hierarchical clustering of differentially expressed gene (DEG)-expression profiles revealed that both lncRNA and mRNA signatures distinguished DCM and control samples with a high degree of accuracy (Figures 2B,C). GO and pathway enrichment analysis were conducted to investigate the functional characteristics of DElncRNAs and DEmRNAs. The top five enriched GO terms and pathways were presented in Figures 2D,E. Upregulated DEmRNAs were related to cellular macromolecule metabolic process in the categories of biological process, and related to transcription regulation in the domain of molecular function. Pathway enrichment analysis showed upregulated DEmRNAs were associated with 5-hydroxytryptamine degradation, circadian clock system, adrenaline and noradrenaline biosynthesis, pyrimidine metabolism, and gamma-aminobutyric acid synthesis, which were closely related to pathophysiology of DCM and heart failure (Figure 2D). Additionally, we noticed downregulated DEmRNAs were highly associated with transmembrane transport, especially ion transmembrane transport (Figure 2D). While the ion channel dysfunction is considered as a main factor for development of DCM (Gigli et al., 2019). Upregulated DElncRNAs seemed to be mostly enriched in segregation of chromosomes during cell division, which involved microtubule organization, spindle body led chromosome movement and localization, and WNT signal transduction (Figure 2E). While downregulated DElncRNAs were involved in peptidase-mediated proteolysis process and glucose metabolism. Only the cellular component results were somewhat counterintuitive (Figure 2E). Briefly, these results suggested that DCM was associated with both DEmRNAs, as well as altered lncRNAs.

### Identification of lncRNA-mRNA Modules Related to DCM Status

**FIGURE 3:**
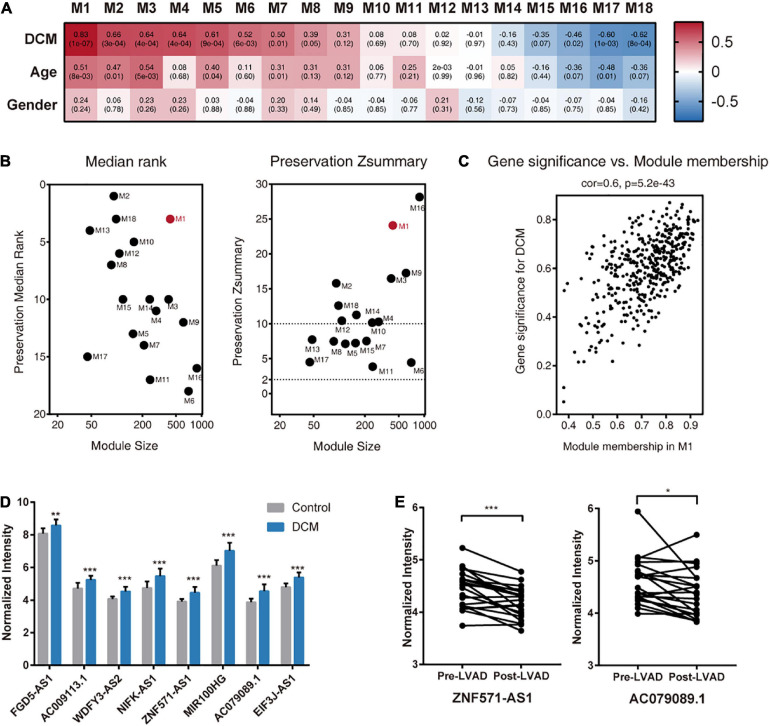
Identification of DCM-associated modules and hub lncRNAs in GSE21610. **(A)** Module-clinical trait association analysis. Each column corresponds to a module, raw to a clinical trait. Each cell contains the corresponding correlation (upper) and *P*-value (lower). The table is color-coded by correlation according to the color legend. **(B)** Module preservation analysis. The median rank (left panel) and Zsummary (right panel) statistics of module preservation of M1–M18 in the validation dataset (GSE1145). **(C)** A scatterplot of gene significance for DCM vs. module membership in the M1. **(D)** Expression of hub lncRNAs in DCM patients and control donors in GSE21610. **(E)** Expression of ZNF571-AS1 and AC079089.1 in DCM patients before and after left ventricular assist device (LVAD) support. All values are mean ± SD. ***P < 0.001; **P < 0.01; *P < 0.05.

To investigate the functional relevance of mRNAs and lncRNAs, we performed WGCNA on GSE21610 and identified 18 gene modules (Figure 3A). To determine a module of clinical interest, we correlated the MEs of each module with traits, such as DCM disease status, age, and sex [ME can be considered the best summary of standardized module expression data (Horvath and Dong, 2008)]. Among these modules, M1 showed the highest association with DCM status (*r* = 0.83, *P* = 1 × 10–7) (Figure 3A). Further investigation of M1 showed it had the highest number of DElncRNAs and DEmRNAs relative to other modules (Supplementary Table 3). Moreover, we found that genes, such as RBM20 (Maatz et al., 2014), FKTN (Murakami et al., 2006), and LAMP2 (Nikolova et al., 2004), previously identified as causative DCM genes were also assigned to M1 (Supplementary Table 4).

To investigate the preservation of M1 in another independent DCM dataset, we used the GSE1145 dataset for validation. Calculation of the Zsummary, a summary preservation statistic that measures both aspects of network density and connectivity preservation, revealed strong evidence (Zsummary >10) that M1 from the GSE21610 dataset was preserved in GSE1145 (Figure 3B). These results indicated that M1 was closely associated with DCM; therefore, we selected M1 for further analysis.

### Identification of Hub lncRNAs

Before identifying hub lncRNAs in M1, we first calculated the MM and gene significance (GS) values of all genes in M1. Because the MM indicates the intramodular connectivity of a gene, it is used as a hub-gene screening strategy, and the GS was used to indicate correlations of the gene with DCM status. The high correlation between GS and MM for genes in M1 demonstrated that genes centrally located in the module were also significantly associated with DCM status (Figure 3C). Therefore, they were natural candidates for further investigation.

We then identified eight centrally located intramodular hub lncRNAs in M1 based on MM values >0.75 (FGD5-AS1, AC009113.1, WDFY3-AS2, NIFK-AS1, ZNF571-AS1, MIR100HG, AC079089.1, and EIF3J-AS1) (Supplementary Table 4). All of the hub lncRNAs were also previously identified DElncRNAs, and there were significant differences in the expression levels of these eight lncRNAs between DCM and control samples (Figure 3D). Next, we applied bootstrap method to assess the reproducibility of these hub lncRNAs screening results. Refer to Methods for detail. Briefly, running the bootstrap with different input gene sets and WGCNA parameters does not change results qualitatively, with marginally acceptable consistency rate of 52.7–62.6% (Supplementary Figure 1).

We determined whether the expression of hub lncRNAs responded to changes in hemodynamic loading conditions. The results showed that ZNF571-AS1 and AC079089.1 expression was significantly reduced in DCM patients with LVAD support (Figure 3E), indicating their potential as useful biomarkers for determining disease status.

We also screened the hub lncRNAs in other modules to identify more possible candidates (Supplementary Table 5). Among these 116 hub lncRNAs, only 13 lncRNAs were DElncRNAs (AL583810.1, FOXP1-IT1, AC005632.2, AC008533.1, L3MBTL4-AS1, AC063926.2, AC026401.3, BASP1-AS1, LINC00967, AL357079.1, TRDN-AS1, AC009318.1, and AP001528.3). All 13 lncRNAs were believed to have potential to serve as biomarkers and drug targets, but existed few flaws compared to the eight hub lncRNAs in M1. For example, many of them were assigned to modules with low preservation, or modules not associated with DCM. Therefore, we mainly focused on the top tier candidates (eight hub lncRNAs in M1) in the following analysis, and treated these 13 lncRNAs as second tier candidates in this study.

### The ceRNA Network Revealed Competing Endogenous Mechanisms of Hub lncRNAs in M1

**FIGURE 4:**
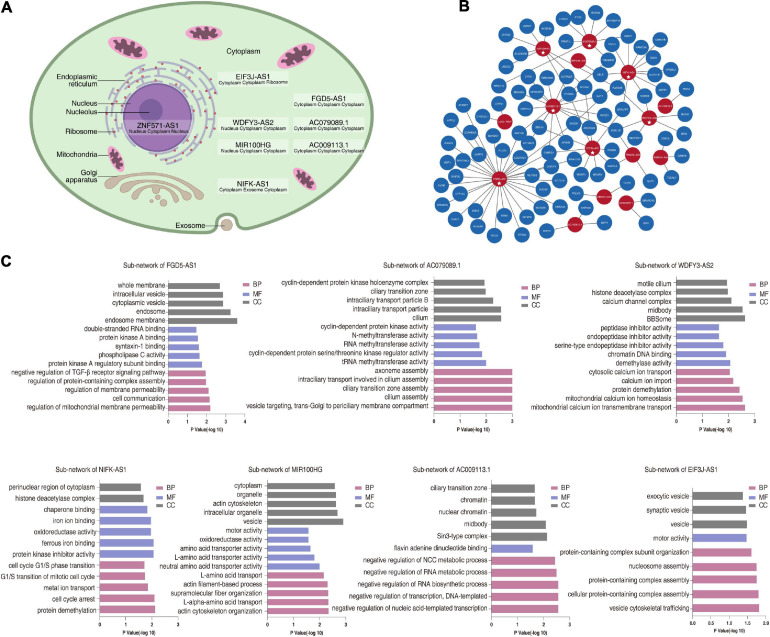
ceRNA network revealed competing endogenous mechanisms of hub lncRNAs in M1. **(A)** Schematic diagram of prediction results of subcellular localization. Blow the gene symbol are the prediction results from DeeplncRNA, lncLocator and iLoc-lncRNA, respectively. **(B)** ceRNA network in M1. The blue nodes represent mRNAs, the red nodes represent lncRNAs, the edges represent the interactions between lncRNAs and mRNAs, and the stars represent hub lncRNAs of M1. **(C)** The top five significantly enriched PANTHER GO terms of genes in each sub-network of hub lncRNAs. NCC, nucleobase-containing compound.

Because lncRNA functions are closely associated with their subcellular localization (Chen, 2016), we investigated where these eight hub lncRNAs were located. According to the majority of the prediction results, ZNF571-AS1 localized to the nucleus while the others localized to the cytoplasm (Figure 4A and Supplementary Table 6).

Based on this result, we hypothesized that these lncRNAs participate in DCM progression by acting as ceRNAs. To test this hypothesis, we used bioinformatics analysis to construct a ceRNA network from M1 based on predicted and experimentally validated miRNA–mRNA/lncRNA regulation and their expression levels. To ensure the reliability of the ceRNA network, lncRNA–mRNA pairs with correlation coefficients <0.7 were removed, resulting in 15 lncRNAs and 92 mRNAs included in the network (Figure 4B and Supplementary Table 7). Consistent with the predictions of subcellular localization, all hub lncRNAs, except ZNF571-AS1, were included in this network. Furthermore, we proposed that the functions of hub lncRNAs may be predicted on the basis of their co-expressing mRNAs. To address this hypothesis, the sub-networks of seven hub lncRNAs were extracted, and GO enrichment analysis was performed (Figure 4C). In general, the functional enrichment results of each sub-network were different from one to another. For example, genes in sub-network of EIF3J-AS1 were mostly enriched in vesicle trafficking, while sub-network of WDFY3-AS2 was associated with calcium ion homeostasis. AC079089.1-associated mRNA were involved in cilium biogenesis. And sub-network of AC009113.1was related to transcription regulation. Finally, among these sub-networks, we also noticed some interesting and promising lncRNA-mRNA pairs. For example, in MIR100HG-LMOD2 pair, the LMOD2 is an actin-binding protein that has recently been implicated in the aberrant cardiac thin filament assembly associated with DCM (Li et al., 2016; Ahrens-Nicklas et al., 2019). In AC009113.1-CRY2 pair, photolyase-like gene CRY2 is essential for maintenance of circadian rhythms in mammalian. While disturbing circadian rhythms disrupts sarcomere structure leading to DCM (Lefta et al., 2012), and adversely affects cardiac contraction and energy consumption (Alibhai et al., 2016). These results indicated that hub lncRNAs in M1 might participate in DCM development partly through ceRNA-related mechanisms.

### External Validation of the Results From Analysis of GSE21610

**FIGURE 5:**
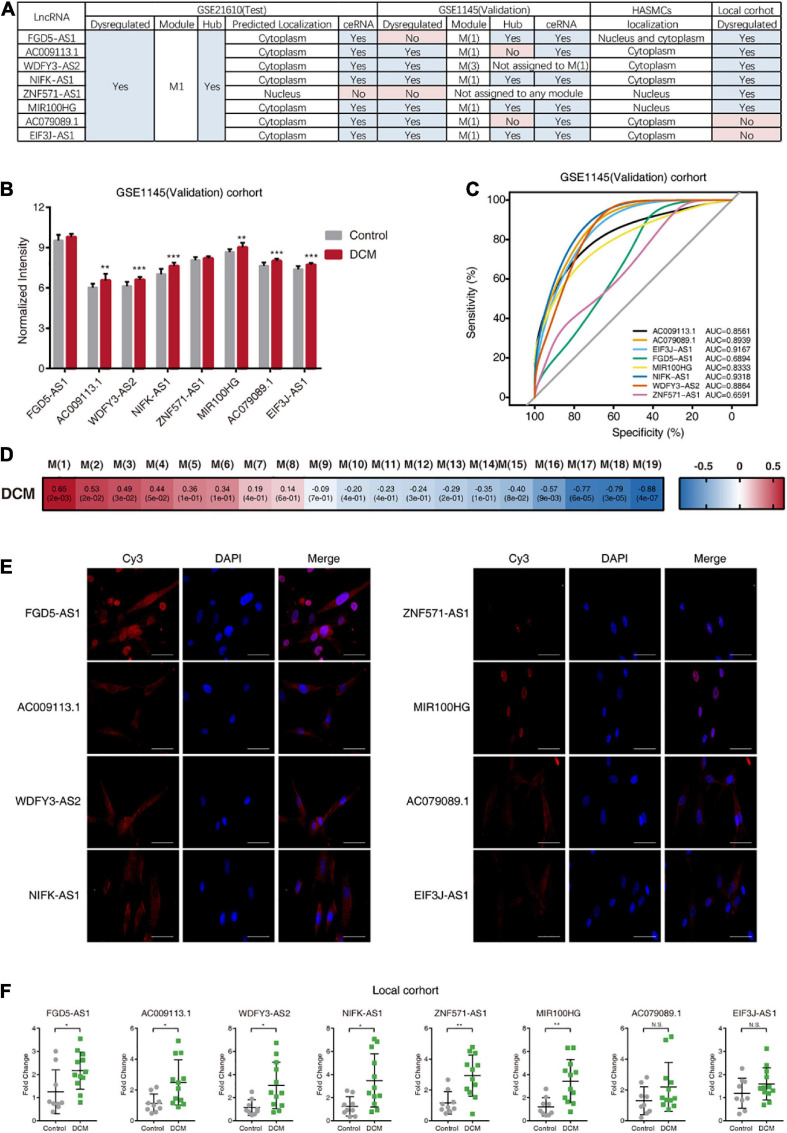
Validation of the results from analysis of GSE21610. **(A)** Overview of the results from different cohorts. “Yes” under the subheading “Hub” means the corresponding gene was the hub lncRNAs in M1 or M(1). “Yes” under the subheading “ceRNA” means the corresponding gene was included in the ceRNA network of M1 or M(1). **(B)** Expression of hub lncRNAs in DCM patients and control donors in GSE1145 (Validation) cohort. **(C)** The receiver operating characteristic (ROC) curve of hub lncRNAs for discriminating between DCM patients and control donors in GSE1145 (Validation) cohort. **(D)** Module-clinical trait association analysis of M(1) of GSE1145 (Validation). **(E)** Fluorescence micrographs of RNA FISH assays, showing subcellular localization patterns of the eight hub lncRNAs in HASMCs. Blue color indicates nuclear staining with DAPI. Scale bars, 50 μm. **(F)** Quantitative RT-PCR for hub lncRNAs expression in DCM patients and control donors in local cohort. AUC, area under the curve.

To confirm the reproducibility of the obtained results, we used a validation dataset (GSE1145) and performed similar bioinformatic analysis on it. First, the expression levels of the eight hub lncRNAs were investigated in the GSE1145, revealing that all hub lncRNAs, except FGD5-AS1 and ZNF571-AS1, were significantly upregulated in DCM patients (Figures 5A,B). Consistently, the results of ROC curve analysis showed that these six hub lncRNAs (AC009113.1, WDFY3-AS2, NIFK-AS1, MIR100HG, AC079089.1, and EIF3J-AS1) displayed high discriminatory power between control donors and DCM patients (Figure 5C). Then we constructed gene modules on GSE1145 using WGCNA (Figure 5D). Of eight hub lncRNAs, six lncRNAs (FGD5-AS1, AC009113.1, NIFK-AS1, MIR100HG, AC079089.1, and EIF3J-AS1) were assigned to a same module, which is M(1) (Supplementary Table 8). Although M(1) was not the most relevant module related to DCM, it still showed high association with DCM status (*r* = 0.65, *P* = 0.002). Further investigation revealed that among these six lncRNAs, four lncRNAs (FGD5-AS1, NIFK-AS1, MIR100HG, EIF3J-AS1) had a MM value exceeding 0.75, which implies these four lncRNAs were the hub nodes both in M1 of GSE21610 and M(1) of GSE1145 (Supplementary Table 8). Moreover, the MM value of the remaining two lncRNAs AC009113.1 and AC079089.1 were also relatively high. Next, we constructed ceRNA network from M(1). All six lncRNAs in M(1) were included in the network (Supplementary Table 9). And this network partly overlapped with the ceRNA network from M1, as they shared 31 same lncRNA-mRNA pairs, which accounted for 28.2% of total number of lncRNA-mRNA pairs in M1 ceRNA networks (Supplementary Table 7). In brief, the consistency of the results between the test and validation datasets demonstrated that these eight hub lncRNAs are essential to DCM disease.

To directly observe the localization of eight hub lncRNAs, we performed RNA FISH in HASMCs (Figure 5E). FGD5-AS1 was detected in both nucleus and cytoplasm; AC009113.1, WDFY3-AS2, NIFK-AS1, AC079089.1, and EIF3J-AS1were predominantly located in the cytoplasm, while ZNF571-AS1 and MIR100HG were mostly nuclear.

In addition, we collected heart tissue from DCM patients and control subjects, and analyzed the expression of hub lncRNAs in this local cohort using quantitative RT-PCR, which showed elevated expression of six hub lncRNAs (FGD5-AS1, AC009113.1, WDFY3-AS2, NIFK-AS1, MIR100HG, and ZNF571-AS1) in heart tissue from DCM patients relative to that in tissue from control subjects (Figure 5F).

## Discussion

In this study, we identified eight candidate lncRNAs associated with DCM and revealed their potential involvement in DCM partly through ceRNAs crosstalk. To the best of our knowledge, this represents the first report identifying these eight lncRNAs as associated with DCM and the first high-throughput study to utilize bioinformatics to investigate how lncRNAs regulate pathogenic coding genes involved in DCM pathogenesis.

### Identification of Hub LncRNAs in M1

To date, several studies have profiled lncRNA transcription in heart tissues of DCM patients utilizing microarray (Li et al., 2018a) and RNA-sequencing (Huang et al., 2019), and identified DElncRNAs as candidate genes for further analysis. Identifying DEGs is critical to transcriptome analysis, because DEGs are often highly associated with disease status. But such candidate gene prioritization method might not consistently identify the driving factors associated with aberrant gene-regulatory networks related to a given disease. In these cases, WGCNA can be used to identify the most connected genes in the pathogenic gene network.

Here, we constructed gene modules using unsupervised clustering, where each module could potentially differ in terms of biological significance. Among these modules, we found that M1 was highly associated with a failing DCM phenotype. Indeed, genes, including RBM20 (Maatz et al., 2014), FKTN (Murakami et al., 2006), and LAMP2 (Nikolova et al., 2004), previously identified as causative DCM genes were assigned to M1. Therefore, we selected M1 for further analysis.

In the context of this study, an ideal result of candidate-gene screening would be their centrality to the gene-network architecture and relationship to DCM status. Our results determined eight regulatory hub lncRNAs in M1 and that were also DEGs, which implied their natural candidacy for further investigation.

Another research of the application of WGCNA to DCM transcriptome data was provided by Zhibing Qiu and his colleagues (Qiu et al., 2019). They identified three DCM-associated lncRNA, which were AC061961.2, LING01-AS1, and RP11-13E1.5. There are two potential reasons for the differences between their results and ours. They filtered genes by differential expression before inputting them to WGCNA, which may invalidate the scale-free topology of gene network and influence the results. Second, they utilized GSAASeqSP (Xiong et al., 2014) to select candidate genes after WGCNA module construction. The core enrichment genes obtained from GSAASeqSP represent genes highly associated with DCM in a certain gene set, rather than the regulatory hub genes in a gene network. Whereas, in the present study we prioritized candidate genes based on their intramodular connectivity, and the eight hub lncRNAs we identified were not the most prominently DEGs. It may explain these hub lncRNAs are unappreciated in Zhibing Qiu’s study (Qiu et al., 2019) as well as others (Li et al., 2018a; Huang et al., 2019).

Indeed, WGCNA is a powerful tool for gathering information about key genes and fundamental drivers of gene expression changes (Silverman et al., 2020). But as recently proposed, one flaw of the gene co-expression network is that it is the proteins encoded by these genes that mediate the effect of genes on diseases. Therefore, it requires to map the hub genes or DEGs to the protein–protein interactions network for optimal interpretation (Silverman et al., 2020).

### Hub LncRNAs Participate in DCM Pathogenesis and Development by Acting as ceRNA

The specific role(s) of lncRNA in DCM development remains vague. Bioinformatics analysis is a screening process that can provide useful insight into lncRNA mechanisms and functions. We constructed a ceRNA network to test the hypothesis that the hub lncRNAs from M1 might participate in DCM progression by acting as ceRNAs based on their subcellular localization. To minimize selection bias, we inserted all lncRNAs in M1 rather than eight hub lncRNAs into ceRNA network construction, with the result showing that all hub lncRNAs, except ZNF571-AS1, remained in the ceRNA network.

Moreover, previous researches have corroborated our ceRNA hypothesis in some way. It is demonstrated that NIFK-AS1 increases the expression of Notch1 by interacting with miR-146a in endometrial cancer (Zhou et al., 2018), and FGD5-AS1 affects periodontitis by regulating the FGD5-AS1/miR-142-3p/SOCS6 axis (Chen et al., 2019).

One limitation of this study was not to include the consideration of miRNA abundance during ceRNA network construction. In other words, we identified shared miRNAs between lncRNAs and mRNAs not based on quantitative miRNA expression profiles, but based on miRNA-target interactions data retrieved from public database. A better way to ensure the lncRNAs regulate mRNA transcription in a miRNA-dependent manner, is proposed by Paola Paci and his/her colleagues. They developed an algorithm to mathematically measures the contributions of miRNAs to lncRNA-mRNA interactions and conducted a seed match analysis (Paci et al., 2014). Another limitation was that we only investigated the possibility of ceRNA crosstalk while cytoplasmic lncRNAs could potentially function through diverse mechanisms, including translation regulation (Yoon et al., 2012), mRNA stability (Gong and Maquat, 2011), and modulating post-transcriptional modifications (Wang et al., 2014). Determination of whether the identified hub lncRNAs in M1 regulate DCM progression through these functions requires further investigation.

### Reproducibility of Results

A common limitation of bioinformatics analysis is poor reproducibility. To increase the reliability of our results, we used a validation DCM dataset and a local cohort to validate our findings. We revealed that M1, the module exhibiting the highest degree of association with DCM, was present in the validation dataset (Zsummary >10). Notably, Zsummary values often depend on the module size and tend to increase as the module size increases (Langfelder et al., 2011). Therefore, we also calculated the medianRank value, which is much less dependent on module size (Langfelder et al., 2011), revealing that M1 ranked second among all modules (Figure 3B), supporting the connectivity patterns among genes in M1 as being reproducible. We then conducted WGCNA and ceRNA network construction on GSE1145 (Validation). And the result showed good overall reproducibility. In addition, qRT-PCR experiments validated expression changes in six of eight hub lncRNAs in DCM patients of a local cohort. These data demonstrated our former results are reproducible among multiple institutions; however, further experimental validation and investigation are required.

## Data Availability Statement

The original contributions presented in the study are included in the article/Supplementary Material, further inquiries can be directed to the corresponding author/s.

## Ethics Statement

The studies involving human participants were reviewed and approved by the Ethics Committee of the First Affiliated Hospital, Zhejiang University School of Medicine. Written informed consent to participate in this study was provided by the participants’ legal guardian/next of kin.

## Author Contributions

Y-XC, S-JH, and JY: conceptualization. S-JH: data curation. X-TS: formal analysis. Y-XC, JD, W-EZ, X-TS, X-YW, and CZ: investigation. Y-XC, JD, X-YW, G-FS, and CZ: methodology. Y-XC, X-TS, NL, and XZ: visualization. Y-XC: writing – original draft. S-JH and JY: writing – review and editing. All authors contributed to the article and approved the submitted version.

## Conflict of Interest

The authors declare that the research was conducted in the absence of any commercial or financial relationships that could be construed as a potential conflict of interest.

## Supplementary Material

Supplementary Figure 1 Visualization of the results of bootstrap methods to assess the robustness of the hub lncRNAs. **(A–C)** we randomly re-sampled 90% of the initial input gene set 100 times followed by weighted gene co-expression network construction; **(D–F)** we used various combinations of parameters to recreate the network 28 times. **(A,D)** Heatmaps show whether corresponding genes remained intramodular hub genes (MM > 0.75) in their modules. Each cell contains the corresponding MM value. **(B,E)** Heatmaps show whether corresponding genes were assigned to the most significant DCM-associated module. Module was ranked according to DCM-correlation coefficient. Each cell contains the ranking number of the module to which corresponding gene was assigned. For example, “1” means the corresponding gene was assigned to the most significant DCM-associated module. **(C,F)** Heatmaps show whether corresponding genes fulfilled both above two criteria. And the red shading is used to illustrate that corresponding genes have met the criteria. The consistency rates are calculated as the ratio of the number of the shaded cells to the total number of cells.

Supplementary Figure 2 Quality Control of candidate datasets. **(A)** Relative log expression (RLE) plots **(B)** Normalized unscaled standard error (NUSE) plots **(C)** Histograms of candidate datasets GSE21610 (Test), GSE1145 (Validation) and GSE19303. And they did not appear to present any quality problems. **(D)** RNA degradation plot of candidate datasets, chips in GSE19303 showed steeper slops, which indicates low RNA quality.

Supplementary Figure 3 Analysis of network scale free topology for various soft-thresholding powers of GSE21610 (Test) (left) and GSE1145 (Validation) (right).
