# Supplementary figures and images for: Identification and Functional Prediction of Long Non-Coding RNAs in Dilated Cardiomyopathy by Bioinformatics Analysis
Source: Front Genet. 2021 Apr 16;12:648111. doi: 10.3389/fgene.2021.648111 (PMC8085533; doi:10.3389/fgene.2021.648111)

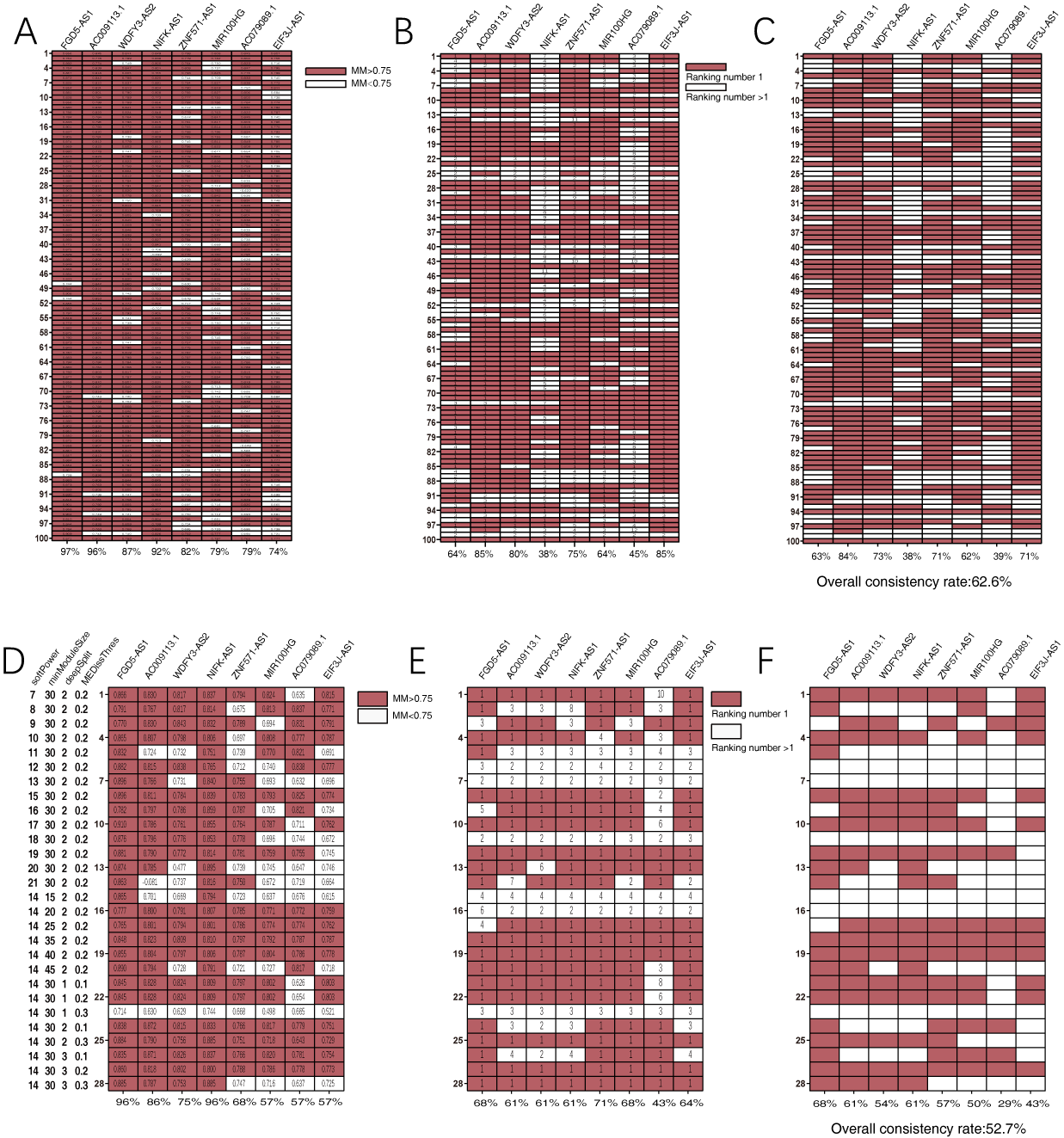

Supplement: Supplementary Figure 1 — Visualization of the results of bootstrap methods to assess the robustness of the hub lncRNAs. (A–C) we randomly re-sampled 90% of the initial input gene set 100 times followed by weighted gene co-expression network construction; (D–F) we used various combinations of parameters to recreate the network 28 times. (A,D) Heatmaps show whether corresponding genes remained intramodular hub genes (MM > 0.75) in their modules. Each cell contains the corresponding MM value. (B,E) Heatmaps show whether corresponding genes were assigned to the most significant DCM-associated module. Module was ranked according to DCM-correlation coefficient. Each cell contains the ranking number of the module to which corresponding gene was assigned. For example, “1” means the corresponding gene was assigned to the most significant DCM-associated module. (C,F) Heatmaps show whether corresponding genes fulfilled both above two criteria. And the red shading is used to illustrate that corresponding genes have met the criteria. The consistency rates are calculated as the ratio of the number of the shaded cells to the total number of cells. [file Image_1.TIF]

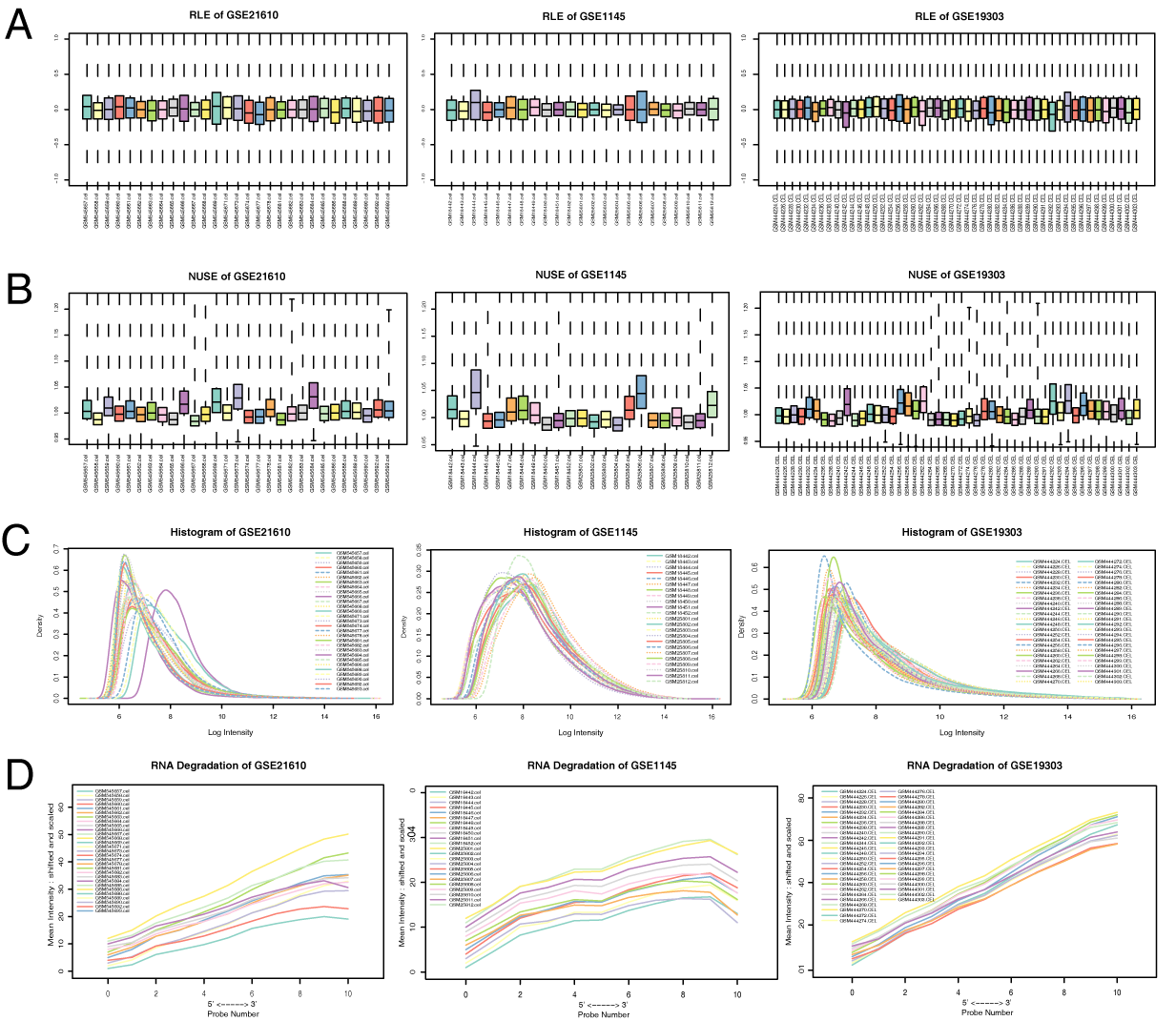

Supplement: Supplementary Figure 2 — Quality Control of candidate datasets. (A) Relative log expression (RLE) plots (B) Normalized unscaled standard error (NUSE) plots (C) Histograms of candidate datasets GSE21610 (Test), GSE1145 (Validation) and GSE19303. And they did not appear to present any quality problems. (D) RNA degradation plot of candidate datasets, chips in GSE19303 showed steeper slops, which indicates low RNA quality. [file Image_2.TIF]

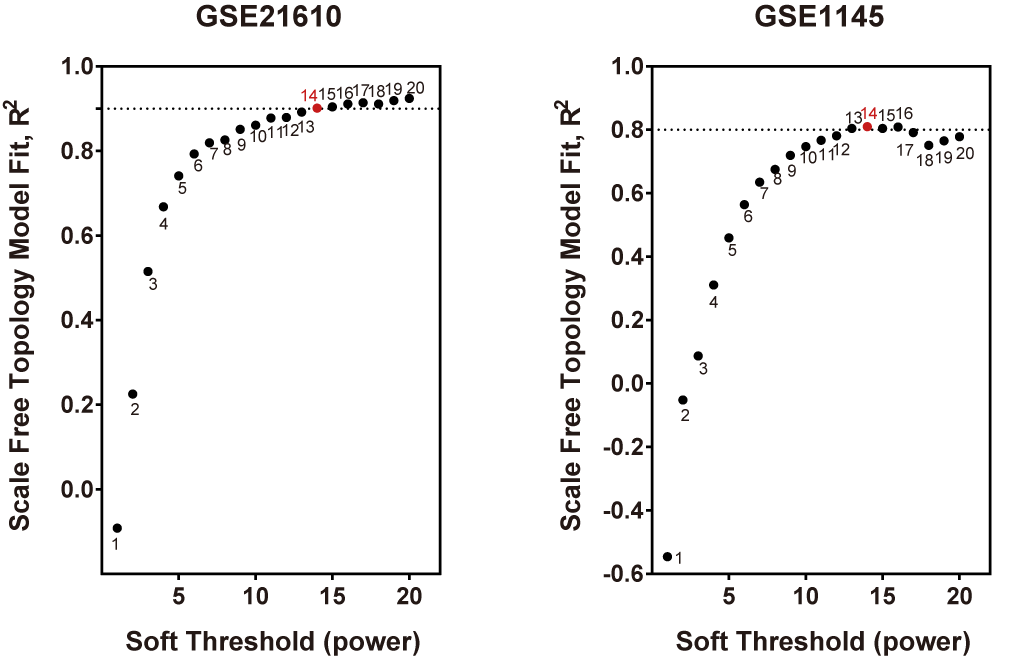

Supplement: Supplementary Figure 3 — Analysis of network scale free topology for various soft-thresholding powers of GSE21610 (Test) (left) and GSE1145 (Validation) (right). [file Image_3.TIF]
