# Supplementary material for: Identification and Functional Prediction of Long Non-Coding RNAs in Dilated Cardiomyopathy by Bioinformatics Analysis
Source: Front Genet. 2021 Apr 16;12:648111. doi: 10.3389/fgene.2021.648111 (PMC8085533; doi:10.3389/fgene.2021.648111)
Supplement: Supplementary file 15 [file Table_12.DOCX]

Supplementary Material

# Supplementary Materials and Methods

## Study Selection Strategy

We systematically searched Gene Expression Omnibus (GEO) database using the keyword “Dilated Cardiomyopathy”. At the time of our analysis (2019-03-01), 1213 GEO datasets were associated with the keyword. Then we filtered the 1213 results by certain constraints (Organism: Homo sapiens(human), Study Type: Expression profiling by array, Entry type: Series), and yielded 34 eligible datasets. Then a detailed investigation was carried out on these 34 datasets to evaluate if the following criterias were fulfilled (Supplementary Table S10):

(a) The subjects of the gene expression experiments were DCM patients. And the number of DCM subjects should exceed eight. Meanwhile, any study that predominantly focused on a certain subtype of DCM should be excluded.

(b) The gene chip platform used in the dataset is unable to annotate lncRNAs due to the incomplete and outdated annotation tables provided by manufacturer. Only after re-annotation, the chip will be able to measure the expression of lncRNAs.

(c)Analyzable raw data CEL files are available in GEO web site.

(d) RNA samples were extracted from human left ventricular tissue.

Eventually we found 6 datasets fulfilling the inclusion criteria, which were GSE21610, GSE1145, GSE79962, GSE42955, GSE19303 and GSE17800. We selected GSE21610 as the test dataset, set aside GSE1145 as a validation dataset.

We did not select GSE79962 or GSE42955 as the test dataset or validation dataset. It is because the microarray chip platform used in GSE79962 and GSE42955 was Affymetrix HuGene-1_0-st. In the Affymetrix HuGene-1_0-st platform, the number of lncRNAs we re-annotated was only 681. Therefore, neither GSE79962 nor GSE42955 was the optimal choice for us to perform further analysis.

We did not select GSE19303 as the test dataset or validation dataset. It is because the quality assessment of these datasets suggested that there was noticeable RNA degradation effect in data files of GSE19303 (slopes>3.4) (Supplementary Figure S2, Supplementary Table S11). Since low-quality RNA may compromise the experimental results, GSE19303 were not considered for further analysis. Meanwhile GSE17800 and GSE19303 are essentially overlapping, as they include the same patients. Therefore, both GSE17800 and GSE19303 were excluded from the analysis.

Both GSE21610 and GSE1145 were found to be of good quality. But in the end, we utilized GSE21610 as test dataset, and GSE1145 as validation dataset, rather than the reverse. It is because we found GSE1145(Validation) is less suitable for WGCNA network construction than GSE21610(Test). More specifically, WGCNA was utilized to construct a scale-free gene network. The scale-free topology ensures the presence of a few node genes (hub genes) with massive links to other genes, rather than all nodes have approximately the same number of links. Soft thresholding power β is an important parameter of gene co-expression network construction. In GSE21610(Test), when the β was set to its optimal value (β=14), we can get a good scale-free topology index (R2>0.9) (Supplementary Figure S3). But in GSE1145(Validation), the highest R2 we can get is just 0.81. As suggested by the author of WGCNA, if the R2 which quantifies the fit of scale-free topology, fails to reach values above 0.8 for reasonable Soft thresholding powers (β<15), the scale-free topology assumption could be invalidated. As WGCNA is an indispensable process for test dataset, we believe GSE21610(Test) is the optimal choice for test dataset selection.

## Quality Control

To assess the quality of candidate datasets, we choose four widely used metrics: the relative log expression (RLE), normalized unscaled standard error (NUSE), histogram and RNA degradation slope. These metrics were calculated using “affy” and “affyPLM” package as described in[1]. RLE plots visualize the deviations from gene medians. The boxes in RLE plots are expected to be centered around 0. NUSE plots provide the information about chip-wise normalized unscaled standard errors obtained for the genes. An aberrant chip on NUSE plot might be indicated by a higher median (>1.05)[2]. The histograms show the log scale intensities if each chip. Bimodal distribution, or spikes at the low or high end of distribution suggests that one has a problem with the chip[1]. As to RNA degradation plots, when RNA degradation occurs, intensities should be significantly elevated at the 3’ end of the probeset when compared to the 5’ end. And a slope value that exceed 3.4 might indicate degradation for HG-U133A chips[1]

# References

1. Bolstad B M, Collin F, Brettschneider J, Simpson K, Cope L,Irizarry R A, et al. Quality Assessment of Affymetrix GeneChip Data. In: Gentleman R., Carey V.J., Huber W., Irizarry R.A. and Dudoit S. (Eds), Bioinformatics and Computational Biology Solutions Using R and Bioconductor. Springer New York, New York, NY, 2005, pp. 33-47.

2. Heber S,Sick B. Quality assessment of Affymetrix GeneChip data. Omics. 2006;10(3):358-368.
